# Effect of Dietary *Clostridium butyricum* Supplementation on Growth Performance, Intestinal Barrier Function, Immune Function, and Microbiota Diversity of Pekin Ducks

**DOI:** 10.3390/ani11092514

**Published:** 2021-08-26

**Authors:** Yanhan Liu, Cun Liu, Keying An, Xiaowei Gong, Zhaofei Xia

**Affiliations:** 1 College of Veterinary Medicine, China Agricultural University, Beijing 100193, China; vetlyh@163.com (Y.L.); s20193050785@cau.edu.cn (K.A.); gxw@cau.edu.cn (X.G.); 2 Shandong Provincial Center for Animal Disease Control, Ji’nan 250100, China; liucun89@163.com

**Keywords:** *C. butyricum*, intestine microbial, Pekin duck, SCFAs, tight junction

## Abstract

**Simple Summary:**

In poultry farming, the use of prophylactic antibiotics can lead to increased resistance, so probiotics are a good alternative. *Clostridium butyricum (C. butyricum)* has been widely used to improve the gut health of animals. Therefore, we carried out the current study of Pekin ducks supplemented with *C. butyricum* for a period of 42 days. Here, we found a clear increase in the growth performance of Pekin ducks supplemented with *C. butyricum*. Moreover, a high level of secretory IgA, IgM, IgG, IL-4, and IL-10 and comparatively higher short-chain fatty acids (SCFAs) and intestinal tight junction changes were found in Pekin ducks supplemented with *C. butyricum*. The gut microbial diversity of Pekin ducks supplemented with *C. butyricum* was clearly different than that of Pekin ducks fed a non-*C. butyricum* diet. In conclusion, our findings suggest that 400 mg/kg *C. butyricum* supplementation improved the intestinal health of Pekin ducks by increasing the α-diversity of intestinal microbiota, enhancing the SCFAs contents, and strengthening the intestinal barrier function and immune systems indicating that 400 mg/kg *C. butyricum* might be a preferable antibiotic alternative for commercial application.

**Abstract:**

*Clostridium butyricum* (*C. butyricum*) is increasingly being used to test the promotion of the gut health of animals. However, the modes of action for such applications for waterfowl remain unclear. Thus, we investigated whether or not intestinal barrier function, immune-related gene expression, and the diversity of the intestinal microbiota in Pekin ducks varied under *C. butyricum* supplementation. A total of 500 ducks were randomly assigned into five treatments supplemented with basal diets containing: either 0 (group Control), 200 (group CB200), 400 (group CB400) and 600 (group CB600) mg/kg *C. butyricum* or 150 mg/kg aureomycin (group A150) for 42 days. In comparison with the control group, *C. butyricum* supplementation enhanced the growth performance and intestinal villus height of Pekin ducks at 42 d. Serum immune indexes and fecal short-chain fatty acids (SCFAs) were all improved at both 21 d and 42 d after *C. butyricum* addition. The *mRNA* expression levels of *Mucin2*, Zonula occludens-1 (*ZO-1*), *Caudin-3*, and *Occludin* increased at 21 d and 42 d and the mRNA expression levels of IL-4 and IL-10 only increased at 42 d after *C. butyricum* addition. Dietary *C. butyricum* also resulted in an increase in the number of diversities of operational taxonomic units (OTUs), and an increase in the α-diversity of intestinal microbiota. The addition of *C. butyricum* altered the composition of the intestinal microbiota from 21 d to 42 d. The relative abundance of *Firmicutes* and *Bacteroidetes* showed little changes among groups; however, the relative abundance of *Firmicutes*/*Bacteroidetes* were found to have been significantly different between the 21 d and 42 d. *C. butyricum* administration improved the intestinal health of Pekin ducks by increasing the diversity of intestinal microbiota, enhancing the SCFAs contents, and strengthening the intestinal barrier function and immune systems. The optimal dietary supplementation dosage was recommended as 400 mg/kg in the diet.

## 1. Introduction

Probiotics have been found to be able to improve digestive function and prevent leaky intestines or intestinal inflammation. *Clostridium butyricum* (*C. butyricum*) is considered a Gram-positive obligate anaerobic probiotic which can produce butyric acid and form endospores, and has been documented as normal intestinal flora of healthy animals [1]. *C. butyricum* can tolerate high bile, high temperature environments, and acidic environments of the digestive tract, thus, it can be used as an effective feed additive [2]. *C. butyricum* can produce bacteriocin, lipoteichoic acid, hydrogen, and these prebiotics can help to improve intestinal anti-oxidation and anti-bacterial functions in animals [3]. *C. butyricum* is able to increase growth performance, alleviate oxidative stress, and strengthen immune function of broiler chickens and cultured shrimps [4,5]. In our own studies, we demonstrated that dietary *C. butyricum* intervention modulated serum lipid metabolism and improved both meat quality as well as fatty acid composition of Pekin ducks [6]. Therefore, *C. butyricum* application could promote growth performance, increase SCFA contents, and improve immune function of the intestine. However, such questions and the effects of *C. butyricum* application on the diversity of intestinal microbiota of waterfowl including for Pekin ducks has not been well researched.

Tight junctions have been considered as the primary apical components of intercellular junctions of intestinal epithelial cells [7]. Zonula occludens-1 (*ZO-1*) was the first intracellular component to be identified as a tight junction in 1986 [8]. Tight junctions can maintain desirable levels of permeability and barrier integrity of intestinal epithelial cells based upon examinations of both healthy and diseased treatment groups [9]. The gut plays a key role in enabling efficient absorption of nutrients due to its large surface area. The gut also includes enormous numbers and a diversity of micro-organisms, such as including opportunistic pathogens [10] which can disrupt intestinal barrier mechanisms and integrity, and induce inflammation through the release of cytokines which further disrupt the intestinal barrier. Hence, it is important for animals to have a healthy gut to limit the release of inflammatory cytokines that disrupt the intestinal barrier [11]. However, some pro-inflammatory cytokines that are known to exist in the intestine are beneficial to the overall integrity of the intestinal barrier and are important in the stimulation of release of anti-microbial substances [12]. For example, interleukin 1 beta (IL-1β) is deemed to damage tight junctions but is known to be able to beneficially stimulate the expression of anti-inflammatory cytokines including IL-4 and IL-10 which strengthen integrity of the intestinal barrier [13]. However, the effects of dietary *C. butyricum* supplementation on tight junctions and anti-inflammatory cytokines in the intestines of Pekin ducks are unknown.

Recent increasing attention has investigated potential positive effects of applied probiotics for gut microbial communities. Several researchers have demonstrated that increased diversity of intestinal microbiota can affect various aspects of animal health including such as via regulation of the gut-liver axis and gut-brain axis [14,15]. Probiotics have been the most commonly applied alternative traditional medicines to regulate intestinal microbiota [16]. *C. butyricum* is thought to help optimize the intestinal microbiota and harmonize intestinal microecology via preventing the proliferation of malignant bacteria [17]. However, whether or not dietary *C. butyricum* supplementation can be used in a positive way to beneficially impact the diversity of intestinal microbiota and immunity in waterfowl including Pekin ducks remains unclear. In addition, short-chain fatty acids (SCFAs) known as the main metabolites of the microorganisms residing within the gastrointestinal tract can further slightly decrease the pH in the acidic environment of the intestine to induce an increase in the growth of probiotic bacteria, and to prevent the invasion of pathogenic bacteria in order to substantially affect gut health [18,19]. Thus, whether or not dietary *C. butyricum* could affect the concentrations of intestinal SCFAs as well as the diversity of microbial community of Pekin ducks remains to explore.

Therefore, the objectives of the present study were to assess the influences of dietary *C. butyricum* supplementation on growth performance, serum immunologic function, intestinal morphology, SCFAs contents, tight junction, and immune-related gene expression in gut, as well as the diversity of intestinal microbiota in Pekin ducks. This study will provide basic knowledge for the potential mechanism of *C. butyricum* in application used to modulate the gut health of Pekin ducks in the practical production.

## 2. Materials and Methods

### 2.1. Ethics Approval and Consent to Participate

All animal-based protocols conducted in this study were approved by China Agricultural University and in accordance with the Guidelines of the Animal Ethical Committee (permit no. CAU20180428-2).

### 2.2. Dietary Treatments and Feeding

We used five hundred 1-day-old male Pekin ducks (local species conventionally domesticated in Beijing, China) which were obtained from a local commercial hatchery. Ducks were raised in an experimental poultry room of China Agricultural University following normal management routines. Ducks were reared in an air-conditioned house in which could gradually modulate room temperature. Ducks were provided feed and clean drinking water ad libitum and 23 h of light and 1 h of darkness each day for 42 d. Ducks were randomly assigned into five treatments each with 5 replicates (20 ducks/replicate). The control group (group Con) was provided a corn-soybean basal diet without antibiotic or growth promoters. Ducks in the 3 experimental groups were provided a corn-soybean basal diet with 200 mg/kg (group CB200), 400 mg/kg (group CB400), and 600 mg/kg (group CB600) *C. butyricum* (2.0 × 10^9^ cfu). Ducks in the fifth treatment group were provided a corn-soybean basal diet with 150 mg/kg (group A150) aureomycin (powder form). The composition and nutrient levels of the basal diets were calculated to meet or exceed the requirements of the National Research Council (NRC, 1994) for the starter (1–21 d) and grower (22–42 d) periods, respectively (Table 1). The probiotic strain *C. butyricum* (powder form, Batch Code. 20170325003) used in this study was purchased from Beijing Shine Biology Technology Co., Ltd., Beijing, China.

The body weight (BW) of all ducks was individually weighed and feed intake was recorded for ducks from every replicate on 21 days and 42 days, respectively. Average daily feed intake (ADFI), average daily gain (ADG), and feed conversion ratio (FCR) were calculated. On day 21 and day 42, five ducks (one per replicate) of each group were randomly selected and used to obtain fresh fecal samples which were rapidly frozen in liquid nitrogen for subsequent bacterial DNA and bacterial 16S rDNA analysis.

### 2.3. Serum Measurements

Blood samples from ten ducks per group were collected from the jugular vein on day 21 and day 42 and were centrifuged at 3000× *g* for 10 min; and then serum was separated and stored at −20 °C for subsequent study. The levels of serum IgA, IgM, and IgG levels were determined using methods for ELISA commercial kits (Nanjing Jiancheng Bioengineering Co., Ltd., Nanjing, China). The concentrations of inflammatory cytokines IL-1β, IL-4, IL-6, IL-10, and tumor necrosis factor-α (TNF-α) were quantified with duck special cytokine/chemokine kits (Nanjing Jiancheng Bioengineering Co., Ltd., Nanjing, China). All measurements were conducted following the manufacturers’ guidelines.

### 2.4. Morphological Observation and Analyses

Ten ducks from each group were euthanized using sodium pentobarbitone (30 mg/kg BW). Distal jejunum (the dividing line between jejunum and ileum is vitelline stalk) segments were fixed in 4% formalin solution for 48 h, dehydrated by a series of alcohol solutions, cleared in xylene, and finally were embedded in paraffin. Sections of annular of 4–5 µm thickness were stained with hematoxylin and eosin (H&E). Intestinal tissue sample sections were scanned utilizing a Leica DMR microscope (Leica Microsystems, Wetzlar, Germany). Under the determination of a treatment-blind viewer, ten well-oriented villus and crypts were selected to evaluate the villus height and crypt depth at magnifications of 50× for each section using the ImagePro Plus Software. The distance of villus height was measured from the top of the villus to the villus-crypt junction; the distance of crypt depth was determined from the villus-crypt junction to the root of the crypt. Then the ratio of villus height to crypt depth (V/C) was determined.

### 2.5. Measurement of Intestinal SCFAs

After animals were euthanized, we sampled fresh cecal contents (approximately 0.5 g) from ten ducks per group which was diluted with a carbonate-phosphate solution. Samples were incubated using an ultrasonic bath for 30 min at room temperature, then were centrifuged at 13,000 rpm for 10 min. The supernatant fraction (500 μL) was collected to a new tube and then filtered through a 0.22 μm filter. SCFAs analysis were conducted by using GC/MC (5975-7890A, Agilent Technologies, Inc., Santa Clare, CA, USA) and followed the guidelines described previously [20]. The areas of peaks for concentrations of acetate, propionate, and butryrate relative to 4-methyl valeric acid were assessed to allow for the quantification of SCFAs.

### 2.6. Gene Expression Measurement

Total RNA was isolated from frozen jejunum tissue without inclusion of any remaining jejunal contents using TRIZOL Reagent (Invitrogen biotechnology Inc., Carlsbad, CA, USA) following the manufacturer’s guidelines. The integrity of the extracted RNA was analyzed in 1.2% agarose electrophoresis. RNA’s concentration was quantified using the Nanodrop 2000 spectrophotometer (NanoDrop Technologies, Wilmington, DE, USA) at 260 nm; and only high-quality RNA samples were selected according to 260/280 nm ratio (1.8–2.0). Total RNA (10 mg) was reverse transcribed to cDNA using the PrimeScriptTM RT reagent kit with gDNA Eraser (Takara, Dalian, China) following the manufacturer’s procedures and stored at −20℃ for further analysis. Real-time quantitative PCR was conducted to measure the levels of expression of tight junction and immune related genes, including Muc2, ZO-1, Claudin-3, Occludin, IL4, IL10, IL6, and IL17 using the SYBR^®^ Premix Ex TaqTM Kit (TaKaRa, Dalian, China) by an ABI QuantStudio 7 Flex Sequence Detection System (Applied Biosystems, Foster City, CA, USA). The glyceraldehyde-3-phosphate dehydrogenase (GAPDH) gene of Anas platyrhynchos was chosen as the reference internal gene and was used to normalize the initial amounts of RNA of each sample. Specificity of PCR products was measured by analysis of the resultant melting curve. All measurements were performed in triplicates for each biological repeat. The relative fold changes of intestinal gene expression were calculated using the 2^−ΔΔCt^ method. The information of the primer sequences for the detections is listed in Table S1.

### 2.7. Gut Microbiota Analysis

Bacterial DNA was extracted from cecal contents by using the QIAamp-DNA Stool Mini Kit (Qiagen, Hilden, Germany) according to the producer’s protocols. The integrity of the extracted DNA was determined in 1.0% agarose gel electrophoresis. The concentration and quality of the extracted DNA were determined using a NanoDrop 2000 spectrophotometer (Thermo Scientific, Wilmington, DE, USA). The hypervariable 3 and 4 (V3–V4) regions of the bacterial 16S rDNA gene were amplified using the extracted metagenomic DNA by PCR utilizing the specific primers of 338F and 806R (338F: 5′-ACTCCTACGGGAGGCAGCA-3′ and 806R: 5′-GGACTACHVGGGTWTCTAAT-3′) following cycles conditions (95 °C for 30 s, followed by 27 cycles at 95 °C for 20 s, 55 °C for 30 s, and 72 °C for 45 s, followed by a final extension step for 10 min at 72 °C). After amplification, the PCR products were visualized on 1.2% agarose gel by electrophoresis and bands of desired sizes (approximately 300 bp) were purified by a QIA quick Gel Extraction Kit (Qiagen, Hilden, Germany). Amplicon libraries were normalized, pooled, and sequenced by an Illumina HiSeq 2500 PE250 Platform (Illumina, San Diego, CA, USA) at Shanghai Majorbio Bio-pharm Technology Co., Ltd. (Shanghai, China).

Sequence reads were assembled using the QIIME Software package. Raw sequence reads were quality controlled through trimming and demultiplexing steps. High-quality sequences were clustered into the same operational taxonomic units (OTUs) with a 97% similarity threshold using UPARSE (version 7.1, http://drive5.com/uparse/, accessed on 12 November 2019). Representative sequences for each OTU were selected for subsequent annotation. For each representative sequence, the GreenGenes reference database was used based on the QIIME platform (http://qiime.org/scripts/assign_taxonomy.html, accessed on 16 December 2019) and the RDP classifier algorithm to annotate taxonomic information. Subsequent analyses of alpha diversity index including Ace, Chao, Shannon and Sobs were calculated using QIIME platform and were visually displayed with R software. Richness and biodiversity were evaluated using measures of alpha diversity. Venn diagram was used to count the number of common and unique OTUs or individual species between groups. According to the taxonomic analyses, community structure composition of different groups at each classification level, such as at the levels of phylum and genus, were determined. Based upon the results obtained from community abundance data, microbiota differences for different groups at each classification level were detected using a rigorous statistical method.

### 2.8. Statistical Analysis

Data were analyzed using SPSS statistical software (SPSS 21.0 Inc., Chicago, IL, USA). The differences among groups in the growth performance, intestinal immune function, tight junction, intestinal morphology, and alpha diversity indices were analyzed by one-way analysis of variance (one-way ANOVA). The relative abundances of phyla and genera among the five groups were analyzed using unpaired Student’s *t*-test. Significance was considered as *p* < 0.05.

## 3. Results

### 3.1. Effect of C. butyricum and Aureomycin on Growth Performance of Pekin Duck

There is no death of ducks during the whole experimental period. From the day 1 to 21, growth performance as indicated by average body weight (ABW), ADG, ADFI, and FCR among the five groups presented no significant differences (*p* > 0.05). However, from 22 d to 42 d, the ABW of groups CB400 and CB600 was enhanced significantly (*p* < 0.05) in comparison with group Con. Results also revealed that the ADG of group CB400 increased significantly (*p* < 0.05) in comparison with group Con; and the FCR of group CB200 and group CB400 reduced significantly (*p* < 0.05) in comparison with results for group Con (Table 2).

### 3.2. Effect of C. butyricum and Aureomycin on Immunologic Function of Pekin Duck

**Figure 1:**
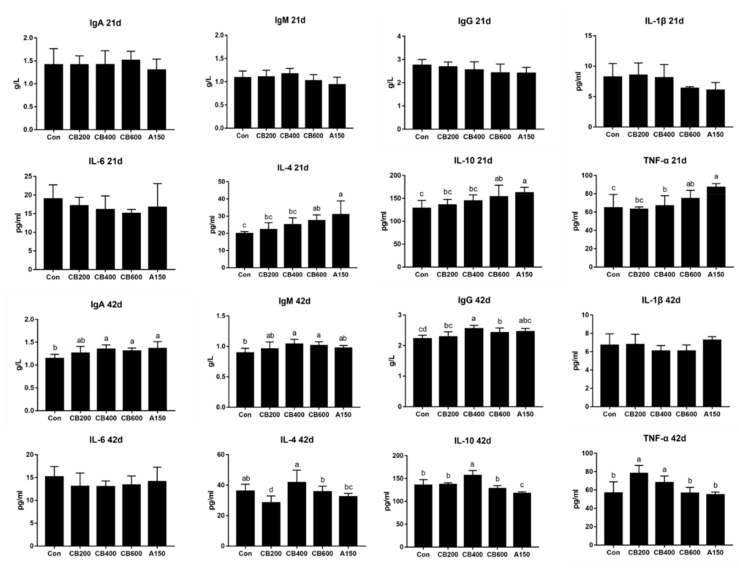
Effects of *C. butyricum* and aureomycin on serum immune parameters of Pekin ducks at 21 d and 42 d, respectively (N = 10 replicates per group). The data were presented as mean ± SD, the superscript different letters indicate that there are significant differences (*p* < 0.05) between any two groups. Con, group Con; CB200, group CB200; CB400, group CB400; CB600, group CB600; A150, group A150.

On day 21, the levels of IgA, IgM, and IgG in serum among the five treatments presented no significant differences (*p* > 0.05). The levels of IL-4 and IL-10 in group CB600 and group A150 had increased significantly (*p* < 0.05) compared to group Con. Likewise, the levels of TNF-α in group A150 enhanced significantly (*p* < 0.05) in comparison with group Con. However, on day 42, the concentrations of IgA, IgM, and IgG in groups CB400 and CB600 increased significantly (*p* < 0.05) in comparison with group Con. The levels of IL-1β and IL-6 presented no significant differences (*p* > 0.05), whereas IL-10 levels in group CB400 were significantly elevated (*p* < 0.05), and TNF-α levels in group CB200 and CB400 enhanced significantly (*p* < 0.05) in comparison with group Con (Figure 1).

### 3.3. Effects of C. butyricum and Aureomycin on Intestinal Morphology of Pekin Duck

**Figure 2:**
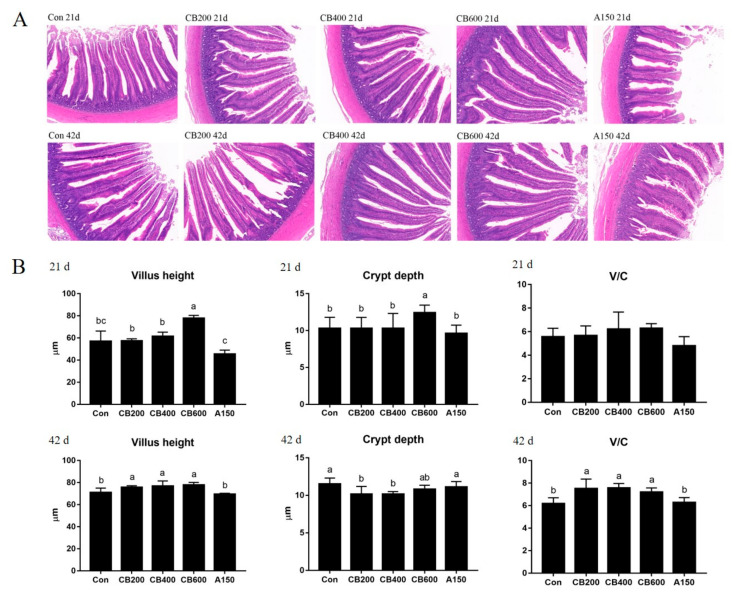
Effects of *C. butyricum* and aureomycin on intestinal morphology of Pekin ducks. (**A**) H&E staining (50×) of intestinal villus. (**B**) The villus height and crypt depth of intestinal villus (N = 10 replicates per group). The data were presented as mean ± SD, the superscript different letters indicate that there are significant differences (*p* < 0.05) between any two groups. V/C, the ratio of villus height to crypt depth. Con, group Con; CB200, group CB200; CB400, group CB400; CB600, group CB600; A150, group A150.

The gut morphology of Pekin ducks fed with *C. butyricum* is shown in Figure 2A. The pathological changes in the ducks in group Con were relatively mild and only a small number of epithelial cells showed dissolution, whereas the *C. butyricum*-treated ducks exhibited a normal appearance and complete structure of the intestinal villus. Aureomycin -treated ducks were found to have presented severe pathological changes with more broken architecture of the villus than was found in the normal group Con. On day 21, enhanced (*p* < 0.05) villus height and crypt depth in group CB600 were observed compared to group Con. The villus height to crypt depth (V/C) ratio showed no significant changes among the five treatments. However, on day 42, greater (*p* < 0.05) villus height and V/C values were obtained in the three *C. butyricum*-treated groups, whereas reduced (*p* < 0.05) crypt depth in groups CB200 and CB400 was recorded when compared to group Con (Figure 2B).

### 3.4. Effect of C. butyricum and Aureomycin on Fecal SCFAs

On day 21, the concentration of propionate in group CB400, CB600, and group A150 was lower (*p* <0.05) whereas the concentrations of butyrate in the *C. butyricum*-treated groups and isovalerate in group CB400 were found to have increased (*p* < 0.05) in comparison with group Con. On day 42, the concentrations of acetate in group CB400 and CB600, propionate in group CB400, butyrate in group CB400 and A150, iso-butyrate and iso-valerate in all groups increased (*p* <0.05) significantly in comparison with group Con (Table 3).

### 3.5. Expression of Tight Junction and Immune Related-Genes in Gut

**Figure 3:**
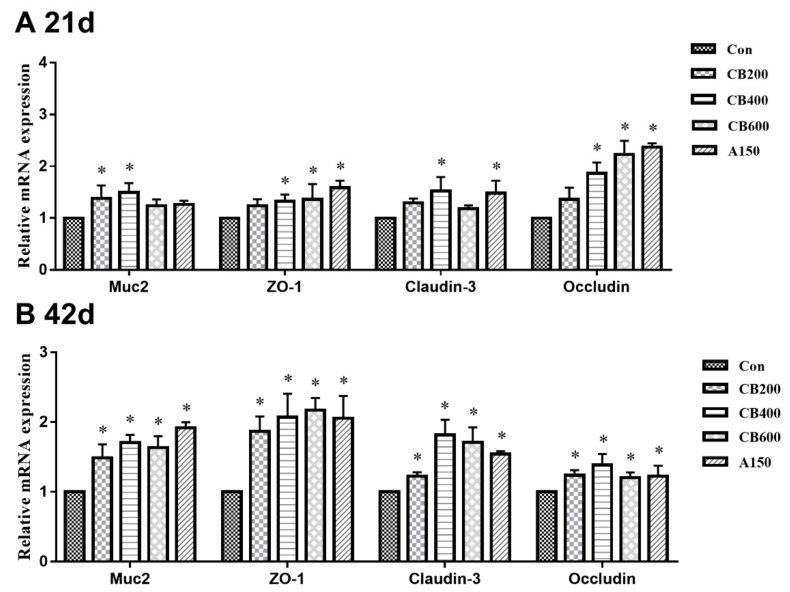
Effects of *C. butyricum* and aureomycin on relative mRNA expression of tight junction protein genes of intestine in Pekin ducks (N = 10 replicates per group). (**A**) The relative mRNA expression of tight junction protein genes at 21 d; (**B**) The relative mRNA expression of tight junction protein genes at 42 d. The superscript * indicate that there are significant differences (*p* < 0.05) compared with the control group. Con, group Con; CB200, group CB200; CB400, group CB400; CB600, group CB600; A150, group A150.

**Figure 4:**
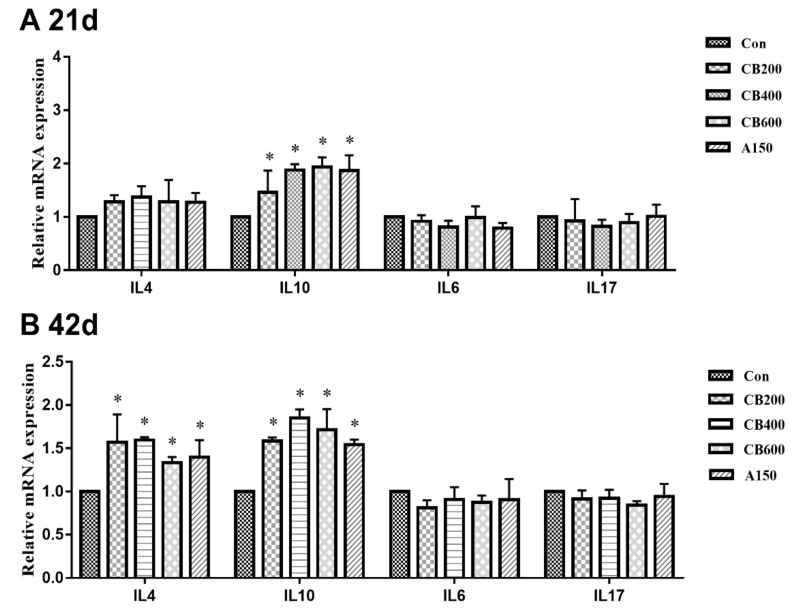
Effects of *C. butyricum* and aureomycin on relative mRNA expression of inflammatory cytokine genes of intestine in Pekin ducks (N = 10 replicates per group). (**A**) The relative mRNA expression of inflammatory cytokine genes at 21 d; (**B**) The relative mRNA expression of inflammatory cytokine genes at 42 d. The superscript * indicate that there are significant differences (*p* < 0.05) compared with the control group. Con, group Con; CB200, group CB200; CB400, group CB400; CB600, group CB600; A150, group A150.

On day 21, in comparison with group Con, the relative levels of mRNA expression of tight junction protein related-gene including Muc2 in group CB200 and CB400, ZO-1 in group CB400, CB600, and A150, Claudin-3 in group CB400 and A150, and Occludin in group CB400, CB600, and A150, were all enhanced (*p* < 0.05) (Figure 3); whereas the relative levels of mRNA expression of immune-related gene such as pro-inflammatory cytokine genes (IL10) increased (*p* < 0.05) and anti-inflammatory cytokine genes (IL6 and IL17) decreased (*p* > 0.05) in the three *C. butyricum*-treated groups (Figure 4). On day 42, in comparison with group Con, the relative levels of mRNA expression of tight junction protein related-gene (Muc2, ZO-1, Claudin-3, and Occludin) and immune-related gene (pro-inflammatory cytokine IL4 and IL10) were also increased (*p* < 0.05) in the three *C. butyricum* -treated groups, whereas the levels of mRNA expression of immune-related gene (anti-inflammatory cytokine IL6 and IL17) were reduced (*p* > 0.05). The mRNA expression levels of tight junction proteins and anti-inflammatory cytokine related-genes were the highest in the group CB400.

### 3.6. Quality of Sequencing Data

**Figure 5:**
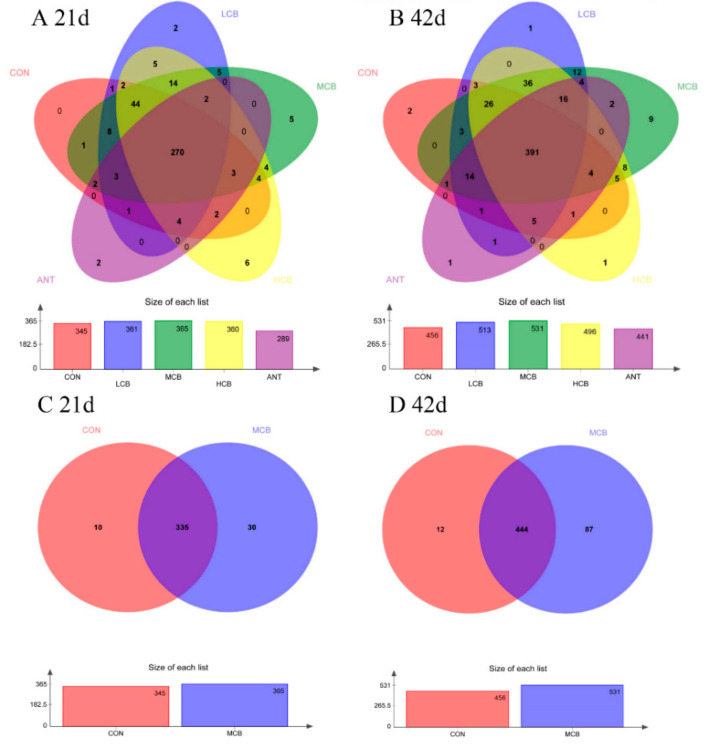
Venn diagram for intestinal microbiota of Pekin ducks (N = 5). Each circle represents a group of samples. The numbers of common bacterial operational taxonomic units (OTUs) are showed in the overlapping part between different circles, while the numbers in the non-overlapping part between different circles represent the number of their respectively unique OTUs in each group. (**A**), Venn diagram of five groups at 21 d; (**B**), Venn diagram of group A and group C at 21 d; (**C**), Venn diagram of five groups at 42 d; (**D**), Venn diagram of group A and group C at 42 d; CON, group Con; LCB, group CB200; MCB, group CB400; HCB, group CB600; ANT, group A150.

16S rDNA gene sequencing of fecal samples was performed to evaluate changes of intestinal bacteria after dietary *C. butyricum* intervention. A total of 1,235,064 and 1,684,981 clean sequences were acquired from fecal samples after quality control, size filtering, and chimera removal at 21 d and 42 d, respectively (Supplementary file 1). The average sequence length was 429 base pairs (bp) with a range of 250 bp to 539 bp at 21 d, and 436 base pairs (bp) with a range of 228 bp to 532 bp at 42 d (Supplementary file 2). The total operational taxonomic units (OTU) numbers which were identified according to >97% sequencing similarity were 390 OTUs and 547 OTUs detected in fecal samples on day 21 and day 42, respectively. The number of OTUs in each group is presented below the Venn diagram on day 21 and day 42, respectively (Figure 5).

**Figure 6:**
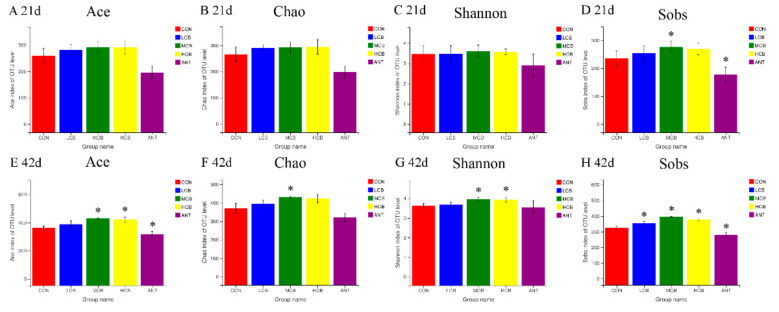
Estimators of α-diversity indices in ceca of Pekin ducks in the five groups at different timepoint. (**A**), Ace index at 21 d; (**B**), Chao index at 21 d; (**C**), Shannon index at 21 d; (**D**), Sobs index at 42 d; (**E**), Ace index at 42 d; (**F**), Chao index at 42 d; (**G**), Shannon index at 42 d; (**H**), Sobs index at 42 d. The superscript * indicates that there are significant differences (*p* < 0.05) compared with the control group. CON, group Con; LCB, group CB200; MCB, group CB400; HCB, group CB600; ANT, group A150.

The Ace index, Chao index, Shannon index, and Sobs index were used to explore the influences of *C. butyricum* on bacterial abundance and diversity. In comparison with group Con (named as CON in Figure 6), the Ace index, Chao index, and Shannon index in *C. butyricum*-treated groups showed no significant differences (*p* > 0.05) at 21 d. The Sobs index in the group CB400 (named as MCB in Figure 6) increased while decreased in the group A150 (named as ANT in Figure 6) significantly (*p* < 0.05) compared with the control group (Figure 6). However, at 42 d, in comparison with the CON, the Ace index in group MCB and group CB600 (named as HCB in Figure 6), the Chao index in group MCB, the Shannon indexes in group MCB and HCB, and the Sobs indexes in three *C. butyricum*-treated groups increased significantly (*p* < 0.05) (Figure 6).

### 3.7. Effect of C. butyricum and Aureomycin on Relative Abundances of Species Structure and Community Composition

**Figure 7:**
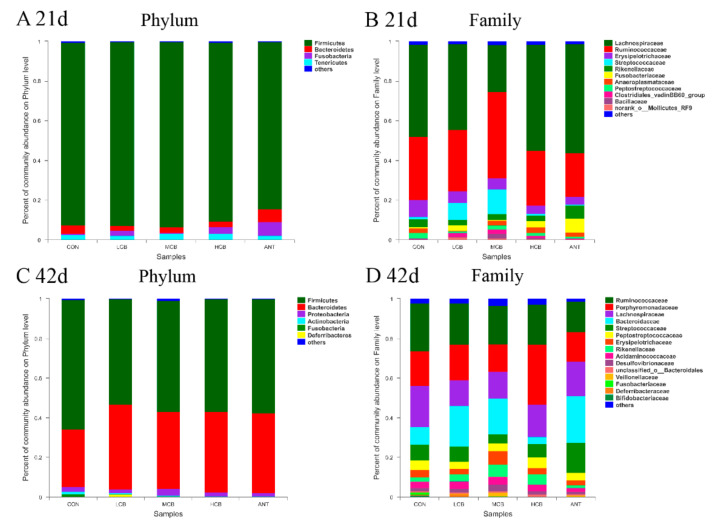
Relative abundance (%) and composition evaluated at the phylum and family levels of the intestinal microbiome of Pekin ducks in the five groups at different timepoint. (**A**), 21 d at phylum level; (**B**), 21 d at family level; (**C**), 42 d at phylum level; (**D**), 42 d at family level. CON, group Con; LCB, group CB200; MCB, group CB400; HCB, group CB600; ANT, group A150.

A total of eight different bacterial phyla were identified on 21 d (Figure 7A). *Firmicutes*, *Bacteroidetes*, *Fusobacteria*, and *Tenericutes* were the four-dominant phylum (relative abundance > 1%) in the five clustered groups. *Firmicutes* was the most abundant phyla in all groups compared to the other phyla, especially in group CB400, as the relative abundance of *Firmicutes* accounted for 92.27% in group Con, 92.65% in group CB200, 93.24% in group CB400, 90.34% in group CB600, and 80.07% in group A150, respectively. The relative abundance of *Bacteroidetes* was found to be 4.04% in group Con, 2.47% in group CB200, 2.87% in group CB400, 2.75% in group CB600, and 6.59% in group A150, respectively. The ratio of *Firmicutes/Bacteroidetes* was found to be 22.84, 37.51, 32.49, 32.85, and 12.15 for the same groups above, respectively. At the family level, *Lachnospiraceae*, *Ruminococcaceae*, *Erysipelotrichaceae*, and *Streptococcaceae* were the primary intestinal bacteria in all of the groups assessed (Figure 7B). The relative abundance of *Lachnospiraceae* was enriched in the groups Con, CB200, CB600, and A150 (relative abundance = 43.00–55.06%) but were apparently weakened in the group CB400 (relative abundance = 23.58%). The relative abundance of *Ruminococcaceae* was enriched in group CB400 (relative abundance = 23.30%) whereas weakened in the other four groups. However, a total of twelve different bacterial phyla were identified on 42 d (Figure 7C). *Firmicutes*, *Bacteroidetes*, *Proteobacteria*, *Actinobacteria*, and *Spirochaetae* were the five dominant phyla (relative abundance > 1%) in the five clustered groups. *Firmicutes* and *Bacteroidetes* were the most abundant two phyla in all groups compared to the other phyla. The relative abundance of *Firmicutes* was 65.21% in group Con, 52.92% in group CB200, 55.85% in group CB400, 56.64% in group CB600, and 57.67% in group A150, respectively. The relative abundance of *Bacteroidetes* was 28.90% in group Con, 42.72% in group CB200, 38.80% in group CB400, 40.52% in group CB600, and 40.17% in group A150, respectively. The ratio of *Firmicutes/Bacteroidetes* was found to be 2.26, 1.24, 1.43, 1.40, and 1.44 for the same groups above, respectively. At the family level, *Bacteroidaceae*, *Lachnospiraceae*, *Prevotellaceae*, *Ruminococcaceae*, *Veillonellaceae*, and *Acidaminococcaceae* were the main intestinal bacteria in all the five groups assessed (Figure 7D). The diversity of species structure and community composition was found to have demonstrated an obvious increased on 42 d at family level.

## 4. Discussion

Numerous studies have demonstrated that an appropriate level of *C. butyricum* administration can increase growth performance in different kinds of animals owing to *C. butyricum* could enhance digestibility of nutrients and energy [5,21,22]. In our study, 400 mg/kg *C. butyricum* can increase ABW of Pekin ducks at day 42, but ADG, ADFI, and FCR were not significantly influenced in the first 21 days of feeding trial. Only ADG and FCR were increased over the second 21 days of feeding trial. These results suggest that positive effects of *C. butyricum* on animal growth were in time-dependent relationship. Our findings are consistent with the above studies.

The strong intestine barrier depends on structural integrity, tight junction proteins, and a stable microbiome [9]. In fact, one of the primary defense mechanisms against invasion is the intestinal epithelial barrier [23]. In order to improve human and animal health, nutritional or pharmacological interventions have been designed to enhance the intestinal epithelial barrier, and such interventions are increasingly being actively sought after by researchers and industry. These positive effects induced by the application of probiotics act as an important role in regulating growth performance and gut health of poultry [24]. Dietary interventions such as probiotics have been found to be able to improve intestinal barrier function via inhibiting inflammation or directly impacting barrier function [25]. Thus, we sought to examine the roles of *C. butyricum* on helping to regulate intestinal immunity and intestinal barrier function as well as the morphological structure of intestinal componentry. To support the above view, an improved intestinal morphology in jejunum was observed in Pekin duck, as evidenced by a significant increase in villus height after dietary 400 mg/kg or 600 mg/kg *C. butyricum* supplementation. In some previous reports, increased intestinal morphological structure was observed in broilers and pigs after probiotic supplementation, which is consistent with ours [21,26,27]. Intestinal villus, as the interface and the link of the host with the environment, plays an important role in maintaining efficient absorption and intestinal barrier integrity [28]. Hence, the increased intestinal morphology manifested an enhancement of intestinal barrier integrity and absorption ability of available nutrients of Pekin ducks in response to *C. butyricum* addition, suggesting that there was better growth performance. As is well-known, *Muc2*, *ZO-1*, *Occludin* and *Claudin-1* are the four primary tight junction proteins to maintain the intestinal barrier function in the small intestine [10]. In previous reports, dietary intervention or inflammation reaction can affect the tight junction proteins [29] and enhanced levels of *mRNA* expression of tight junction proteins including *Occludin* and *ZO-1* can reduce inflammatory stimulus [30]. Our results accordingly demonstrated that *C. butyricum* addition improved the intestinal epithelial barrier function by increasing the mRNA levels of tight junction protein expression. The inflammatory stimuli and other endogenous cytokines were directly to have been directly weakened due to increases in the expression and localization of tight junction proteins which ultimately reinforced the intestinal barrier.

SCFAs are known as the main end products metabolized by microorganisms in the guts of animals [31]. Butyrate has been reported as one of the major SCFAs, and has accordingly been denoted to have a wide range of biological effects. For example, butyrate can inhibit the growth of harmful bacteria, regulate immunity, and change the community composition of gut microbiota [32]. We found that the contents of butyrate in three *C. butyricum*-treated groups at 21 d and in 400 mg/kg and 600 mg/kg *C. butyricum*-treated groups at 42 d enhanced significantly. Dietary supplementation of butyrate in poultry can improve growth performance, carcass yield, intestinal immunity function, and inhibit infection of *Salmonella* enteritidis [33]. Likewise, butyrate was proved to decrease the expression of pro-inflammatory (IL-1β, IL-6, and IFN-α) and increase the expression of anti-inflammatory cytokine (IL-10) genes [34]. These previous researches are in agreement with our results.

Cytokines play a vital role in maintaining the host immune system and intestinal immunity to resist pathogen invasion and modulating the immunity response appropriately [35]. The immune system of poultry gastrointestinal tract can be affected by the probiotics intervention [36]. Probiotics intervention beneficially reinforces the intestinal immunity accompanied by decreased pro-inflammatory cytokines, such as IL-1β, IL-6, and TNF-α and increased anti-inflammatory cytokines, such as IL-4, and IL-10. Increasing the production of anti-inflammatory cytokines might have undiscovered additional underlying advantages in inhibiting intestinal mucosal inflammation [37]. *C. butyricum* can bring a series of microbial activities that stimulate innate immune cells by enhancing immunity response and reducing the production of pro-inflammatory cytokines (IL-1β, IL-6, TNF-α and so on) [38]. In our study, the contents of IgA, IgG, and IgM were not affected by *C. butyricum* treatment at 21 d while increased after 400 mg/kg and 600 mg/kg *C. butyricum* intervention at 42 d. IL-4 and IL-10, which can inhibit the production of pro-inflammatory cytokines, are generated by Th2 cells via *C. butyricum*-stimulated signal transduction of GPR109a [39]. Notably, 400 mg/kg *C. butyricum* supplementation had enhanced the production of IL-10 both in serum at 21 d and gut at 21 d and 42 d suggesting that *C. butyricum* could improve the response of anti-inflammatory cytokines to some extent in Pekin ducks. IL-10 acted an important role in maintaining the production of mucins in intestinal goblet cells, and the mucins can positively reinforce intestinal barrier function [40]. Thereby, the improvement of IL-10 production implied that 400 mg/kg *C. butyricum* likely played a role in the initial protective barrier function of the intestine.

The influences of gut microbiota on growth performance and body health in poultry, for instance digestion and nutrient absorption, immune response, as well as protection of animals against pathogens, have become increasingly noticeable [41]. The diversity of intestinal microbiota is the foundation for digestion and nutrient absorption, the intestinal physiological functions, and the development of the immune system [42]. Previous studies demonstrated that probiotics can regulate intestine microbiota via a competitive exclusion process wherein decreasing the growth of harmful bacteria and increasing the growth of favorable bacteria [43,44,45]. In our current study, an increased richness and biodiversity were found in the intestinal microbial community when Pekin ducks were supplemented with 400 mg/kg *C. butyricum* via OTUs determinations and α-diversity analysis. Further, obvious differences were noted between the *C. butyricum*-treated groups and the control group, suggesting that *C. butyricum* supplementation, especially at a moderate dose (400 mg/kg), enhanced the abundance and diversity of intestine microbiota in Pekin ducks. The Venn diagram based on OTUs analysis also supported the above-mentioned results, which revealed that the ducks fed with 400 mg/kg dietary *C. butyricum* displayed the largest number of unique bacterial OTUs in the intestinal microbiota.

The *Firmicutes* and *Bacteroidetes* dominated the intestinal composition of Pekin ducks at all growth stages which is consistent with previous studies of other animals [27,46]. The results of taxonomic composition comparisons demonstrated an enrichment of the phyla *Firmicutes* and *Bacteroidetes* in the intestine after *C. butyricum* addition. A great number of studies have confirmed probiotic effects of *Bacteroidetes* and *Firmicutes*, which seemingly act as important players in polysaccharide decomposition, and consequently, contribute to improved nutrient utilization, increased immune system development, and help to maintain intestinal microecological balance [47,48]. Notably, the relative abundance of *Firmicutes* and feed utilization presented a positive correlation as we found that the decreased feed conversion ratio of Pekin ducks was accompanied by the enhanced relative abundance of *Firmicutes*. On day 21, at phyla level, approximately 90% of the relative abundant phyla were *Firmicutes* in the intestine of Pekin ducks fed diet with 400 mg/kg *C. butyricum*. This finding may be associated with those younger animals need more intestinal bacteria members belonged to *Firmicutes* for digestion and absorbance of nutrition, for instant *Lachnospiraceae*, *Ruminococcaceae*, *Erysipelotrichaceae*, and *Streptococcaceae*. As Pekin ducks aged, the percentages of *Firmicutes* phyla were reduced, while the population of *Bacteroidetes* phyla was increased in the gut with continuous dietary *C. butyricum* intervention. Therefore, the reduction *in Firmicutes* phyla and the increase in *Bacteroidetes* phyla played a critical role in improving feed efficiency and intestinal structure of ducks with 400 mg/kg *C. butyricum* supplementation. These results were in agreement with a previous report in which *C. butyricum* can enhance the relative abundance of *Bacteroidetes* in weaned pigs [49]. In another study, the dominant functions of intestine microbiota, such as *Firmicutes* and *Bacteroidetes* as well as their interactions, can support the need to further explore a special probiotic that can be best used and can guide us to apply the special probiotics to achieve the anticipated goals for keepers of livestock and breeders.

In addition to our findings, 400 mg/kg *C. butyricum* supplementation presented better regulations on the relative abundance of microbiota communities at the family level form 21 d to 42 d than 200 mg/kg or 600 mg/kg *C. butyricum* supplementation. *Bacteroidaceae* was identified as a beneficial microbe that regulated gut health and helped to absorb polysaccharide from feed which can stimulate the proliferation of *Lactobacillus* [33]. Increased abundances of *Bacteroidaceae* and *Ruminococcaceae* were identified with 400 mg/kg *C. butyricum* intervention. *Ruminococcaceae* was proved to be involved with the degradation of cellulose and hemicelluloses in the intestine [50]. Hence, it can be assumed that the enhanced abundance of *Bacteroidaceae* may be associated with improved intestinal immunity of Pekin ducks. Thus, it could be speculated that 400 mg/kg *C. butyricum* addition better modulates the composition and abundance of favorable and pernicious bacteria, which can improve the intestinal health of ducks.

## 5. Conclusions

In summary, the current study demonstrated that dietary supplementation with *C. butyricum* improved growth performance, host immunity, the concentration of SCFAs, and intestinal tight junctions as well as enhanced abundance and diversity of gut microbiota of Pekin ducks, and the optimum dosage of *C. butyricum* is 400 mg/kg for ducks in the practical production. Further studies can focus on the development of new intervention strategies of probiotics to modulate the gut microbiota to improve the growth performance and productivity of waterfowl.

## Figures and Tables

**Table 1:** Composition and nutrient levels of the basal diets (air-dry basis).

Items	1–21 d	22–42 d
Ingredients (%)		
Corn	56.00	60.24
Soybean meal	32.69	24.67
Wheat middling	5.00	9.00
Soybean oil	2.10	1.80
Phytases	0.02	0.02
Dicalcium phosphate	1.00	1.60
Limestone	1.50	1.20
DL-Methionine	0.15	0.12
L-Lysine	0.20	0.10
Vitamin premix ^1^	0.02	0.02
Trace mineral premix ^2^	0.20	0.20
NaCl	0.35	0.30
Choline chloride (50%)	0.24	0.20
Ethoxyquin (33%)	0.03	0.03
Bentonite	0.50	0.50
Total	100	100
Nutrient levels ^3^ (%)		
AME (MJ/kg)	12.31	12.53
Crude protein (%)	19.52	16.83
Lysine (%)	1.12	0.87
Methionine (%)	0.46	0.39
Calcium (%)	0.88	0.89
Available phosphorus (%)	0.29	0.39
Total phosphorus (%)	0.54	0.62
Methionine + Cysteine (%)	0.79	0.69

^1^ The vitamin premix provided the following per kilogram of diet: vitamin A, 12,500 IU; vitamin D_3_, 3500 IU; vitamin E, 20 IU; vitamin K_3_, 2.65 mg; thiamin, 2.00 mg; riboflavin, 6.00 mg; pyridoxin, 3.00 mg; VB_12_, 0.025 mg; biotin, 0.0325 mg; folic acid, 12.00 mg; pantothenic acid, 50 mg; nicotinic acid, 50.00 mg. ^2^ The mineral premix provided the following per kg of diet: Cu, 6 mg; Fe, 80 mg; Zn, 40 mg; Mn, 100 mg; Se, 0.15mg; I, 0.35 mg. ^3^ Calculated values.

**Table 2:** Effects of *Clostridium butyricum* and aureomycin on growth performance of Pekin ducks ^1^.

Items ^2^	Group Con	Group CB200	Group CB400	Group CB600	Group A150	*SEM* ^3^	*p* Value
1–21 d							
ABW (kg)	1.17	1.17	1.26	1.17	1.07	0.02	0.062
ADG (g/d)	55.98	54.04	56.21	53.95	53.42	0.58	0.439
ADFI (g/d)	90.62	86.36	85.46	89.24	86.16	1.23	0.651
FCR	1.62	1.61	1.52	1.66	1.62	0.03	0.753
22–42 d							
ABW (kg)	2.88 ^b^	2.98 ^b^	3.18 ^a^	3.15 ^a^	2.94 ^b^	0.03	0.009
ADG (g/d)	155.09 ^b^	157.19 ^b^	162.75 ^a^	155.43 ^b^	155.31 ^b^	0.92	0.028
ADFI (g/d)	246.96	236.39	244.32	244.85	243.82	2.08	0.584
FCR	1.59 ^a^	1.50 ^b^	1.50 ^b^	1.58 ^a^	1.57 ^a^	0.01	0.029

^1^ Means with different superscript letters indicate that there are significant differences (*p* < 0.05) between any two groups in the same row. ^2^ ABW: average body weight, ADG: average daily gain, ADFI: average daily feed intake, FCR: feed conversion ratio, Group Con: the control group, Group A150: the aureomycin group. ^3^ Total *SEM* (N = 10).

**Table 3:** Influence of *Clostridium butyricum* and aureomycin supplementation on short-chain fatty acids concentrations of Pekin ducks ^1^ (μg/g).

Items	Group Con	Group CB200	Group CB400	Group CB600	Group A150	*SEM* ^2^	*p* Value
21 day							
Acetate	526.61	517.34	540.61	527.64	504.90	4.60	0.136
Propionate	283.37 ^a^	270.80 ^ac^	245.50 ^bc^	251.82 ^bc^	211.23 ^d^	7.54	0.004
Iso-butyrate	122.81	128.40	137.27	128.32	126.84	1.67	0.056
Butyrate	1360.59 ^d^	1513.37 ^c^	1560.94 ^a^	1556.94 ^ab^	1539.56 ^ab^	20.23	<0.001
Iso-valerate	42.74 ^c^	51.38 ^a^	49.65 ^ab^	45.98 ^bc^	46.14 ^bc^	0.95	0.006
Valerate	62.60	60.18	61.08	57.48	58.97	1.01	0.619
42 day							
Acetate	647.54 ^b^	617.41 ^b^	780.75 ^a^	777.65 ^a^	681.57 ^b^	21.21	0.037
Propionate	283.38 ^ab^	297.46 ^ab^	352.17 ^a^	255.15 ^ab^	218.89 ^b^	4.72	0.022
Iso-butyrate	124.48 ^c^	145.06 ^ab^	157.50 ^ab^	165.00 ^a^	144.84 ^b^	4.40	0.011
Butyrate	1773.96 ^bc^	1909.93 ^ab^	2070.21 ^a^	2000.27 ^a^	1736.23 ^bc^	39.73	0.006
Iso-valerate	48.74 ^b^	74.71 ^a^	87.98 ^a^	89.31 ^a^	89.41 ^a^	5.21	0.024
Valerate	85.93	126.84	124.41	137.48	112.30	6.96	0.152

^1^ Means with different superscript letters indicate that there are significant differences (*p* < 0.05) between any two groups in the same row, Group Con: the control group, Group A150: the aureomycin group. ^2^ Total *SEM* (N = 10).

## Data Availability

Data sharing is not applicable.

## Supplementary Materials

The following are available online at https://www.mdpi.com/article/10.3390/ani11092514/s1, Supplementary file 1: Information of clean reads, Supplementary file 2: Quality control data of each sample, Table S1: Information of primers used in qRT-PCR.
