# Supplementary material for: Effect of Dietary Clostridium butyricum Supplementation on Growth Performance, Intestinal Barrier Function, Immune Function, and Microbiota Diversity of Pekin Ducks
Source: Animals (Basel). 2021 Aug 26;11(9):2514. doi: 10.3390/ani11092514 (PMC8471152; doi:10.3390/ani11092514)
Supplement: Supplementary file 1 [file animals-11-02514-s001.zip › Table S1 Information of primers used in qRT-PCR.pdf]

Table S1 Primers information used for qRT-PCR in this study

| Gene | Primer sequence (5' to 3') | Amplicon size（bp） |
|---|---|---|
| Muc2-F | AGTTCTTGCCTAATTCCTCAGTCT | 146 |
| Muc2-R | TTGCCGTTCATATCCAGGTTCA | |
| ZO-1-F | ACCACCACCTCTTCACAACTAC | 128 |
| ZO-1-R | ACCATCTGCCTTGCCTTCTG | |
| Claudin-3-F | GGCGTCATCTTCCTGCTCTC | 115 |
| Claudin-3-R | GCTCCCTCTTCTGCGATTCAA | |
| Occludin-F | GCAGGATGTGGCAGAGGAATA | 136 |
| Occludin-R | CTTGTCGTAGTCGCTCACCAT | |
| IL-4-F | CAATGAGACAGGCACCGACAT | 219 |
| IL-4-R | GCTACTCGTTGGAGGGTTCTG | |
| IL-10-F | GAACGAGAACGGCATCTACAAG | 86 |
| IL-10-R | TCCTCCTCTTCATCAGCAAGTATT | |
| IL-6-F | AAGTTCACCGTCTGCGAGAA | 78 |
| IL-6-R | GTCTTCCTCCGTCACCTTGG | |
| IL-17-F | AGCCTCTTCAAGCAAGCAGAT | 207 |
| IL-17-R | GCACTCAGCATCAGCAATCAC | |
| GAPDH-F | GTAGTGAAGGCTGCTGCTGAT | 103 |
| GAPDH-R | AGGTGGAGGAATGGCTGTCA | |
